# Analysing students’ environmental awareness profile using strategic environmental assessment

**DOI:** 10.12688/f1000research.51523.2

**Published:** 2021-07-21

**Authors:** Ahmad Khoiri, Widha Sunarno, Sajidan Sajidan, Sukarmin Sukarmin

**Affiliations:** 1 Department of Natural Science Education, Universitas Sebelas Maret, Surakarta, Indonesia; 2 Department of Physics Education, Universitas Sains Al Qur’an, Central Java, Indonesia; 3 Department of Physics Education, Universitas Sebelas Maret, Surakarta, Indonesia; 4 Department of Biology Education, Universitas Sebelas Maret, Surakarta, Indonesia

**Keywords:** Confirmatory Factor Analysis, Environmental Awareness, Ethnoscience, Strategic Environmental Assessment, and Socioscientific Issues

## Abstract

**Background: **Environmental awareness (EA) is a part of character education ignored by most students. This indifference tends to affect other students’ by not only in protecting and preserving the current environment but also in preventing and repairing the damage that occurs in the environment. This research analyses students' EA profile, based on the findings of LISREL 8.8 Confirmatory Factor Analysis.

**Methods: **Research subjects included 131 students from Senior High School State 1 Selomerto Wonosobo, Central Java Province, Indonesia. Based on the Slovin formula, the number of samples is representative of the total population (N: 185; error tolerance: 0.05). Data were collected through non-test questionnaires and observation of Strategic Environmental Awareness (SEA). Quantitative descriptive data analysis on EA indicators (Care, Curiosity, Critical, Dependability, Responsibility, and Local Wisdom).

**Results: **The EA profile of high school students was categorised sufficiently. This revealed the following results: a) the SEA instrument is effective in identifying students’ awareness about current environmental issues and meets model fit criteria (P-value 0.25>0.05; RMSEA 0.095; NFI 0.67); b) the SEA instrument is valid and reliable in accurately determining students’ EA profile; c) although the Responsibility profile was significant (t >1.96), other variables did not meet this significance criteria (EA 1a: ‘Care towards environmental damage’ under the Care profile; EA 3b: ‘Contributing towards preserving the environment’ under the Critical profile; and EA 6b: ‘Aware of local potentials’ under the Local Wisdom profile); d) evaluation of the expected changes in SEA is modified through an ethnoscience approach and the socioscientific issues strategy.

**Conclusions: **Students’ lack of awareness of the environment and understanding of their regional potential fails to contribute towards creating a sustainable environment. Profile analysis in exploring attitudes, values, and ethics towards the environment are important, as it helps recognize students’ behaviour.

## Introduction

Human life cannot be separated from interactions with the environment. Rapid improvement of technology in various fields has left negative impacts on the environment. One such negative impact is environmental damage, which has led to decreased environmental quality. Hence the degradation of environmental conditions can worsen the development of science education. In addition, resources for environmental science learning are still limited. Despite being close to the surrounding environment, science learning is separated from its natural sources. Environmental issues remain under-studied and under-utilized in science learning. ^1, 2^ For instance, how students study the environment has not been integrated into science learning. There is lack of sufficient action to protect the environment, such as taking responsibility, exploring the local wisdom in a particular area, and maintaining and managing the environment. ^3, 4^ Taking care of the environment is an attitude that strives to prevent natural environmental damage, and is the best way to restore environmental damage.

Based on the results of a preliminary survey of 15 teachers and 256 high school students in Wonosobo District, Central Java, Indonesia in March 2020, ^5^ it is observed that schools have shown 75% progress in policies towards environmental awareness, 62% progress in terms of promoting curriculum-based environmental studies, 56% participatory-based development of environmental activities, and 55% management of environmentally friendly school support facilities. That research identified a low implementation of environment-based learning, resulting in a lack of environmental character for students. ^5, 6^

Environmental care, as it has been taught in school, refers to definitions, terms, concepts, and knowledge about the natural environment. The absence of the implementation of contextual learning can limit meaningful experiences for students. Environmental care can be implemented if students develop relevant habits at home, at school, and in the community. This is the best way for schools to build environmentally friendly attitudes among students, that is, by integrating the environment into materials, methods, media, learning resources, and assessment. Hopefully, learning, combined with environmental care, can stir students’ concern for the environment and nature. ^7^

Students’ knowledge of the environment has been positively associated with their environmental care behaviour, which can abate environmental damage. Nurwidodo *et al.* ^6^ stated that students’ environmental care is low because there is a lack of intention to understand and study environmental problems. Therefore, teachers should facilitate students’ understanding of environmental issues in order to motivate them to addresses these problems. ^8^ However, teachers only focus on students’ academic performance and not on how students try to keep the school environment clean. ^9^ Therefore, strategic environmental awareness (SEA) analysis is essential so that students’ positive attitude towards environmental care can provide solutions to environmental problems.

Schools should use an effective instrument to communicate about environmental issues, since environmental care is a crucial issue, especially for students. Learning associated with environmental care can enhance students’ awareness of their environment and surroundings. Creating a healthy ecological system requires long-term efforts. ^10^ This is in congruence with Rahardjo’s ^11^ study, which showed that learning should focus on promoting students’ positive attitude towards the environment. ^12^

SEA assesses environmental care and measures the environment awareness (EA) of students. ^1, 13, 14^ Effective instruments should be valid and reliable. ^15, 16^ Therefore, SEA instruments are required to measure EA skills. EA indicators are determined based on healthy environmental problems, policies in preserving the environment, and the long-term SEA program. ^13^ EA is incorporated in teaching Physics, including Care, Protection, and Conservation Component indicators. ^1^

Based on environmental care, ^17, 18^ EA assumes conscious thought in managing the environment is a factor in protecting and maintaining the environment. ^19, 6, 20^ This does not only demonstrate the understanding of the natural environment but also the attitude, value, and, skills required to address environmental issues. ^21, 22^ Environmental education enhances people’s knowledge and awareness about the environment and induces environmental care behaviour. ^23^ Phan Hoang and Kato ^24^ and Mei *et al.* ^25^ explain that knowledge and commitment are required to instigate EA. Students must gain knowledge and understanding of the fundamental environmental problems, which will initiate a change in attitude and awareness in their social life namely interactions with other humans and the surrounding environment. ^20, 26, 27^ The impact of the students’ low environmental care can be assessed by evaluating the sustainable impact on condition, health, comfort and environmental benefits. ^28– 30^

In Indonesia, this attitude of caring for the environment has become integrated into academic culture. ^6^ The Adiwiyata program in schools seems to be separated from the environmental-based education curriculum, even though EA should be integrated into the students’ learning process of environment friendly characteristics. The current research focuses on the importance of analysing the profile of students’ EA in facing challenges of globalisation. The profile analysis is adapted from the SEA with a holistic role and paradigm aimed at not only developing a caring attitude, maintaining cleanliness, and preserving the environment, but also making a real contribution through policy recommendations, planning, and environmental programs for sustainable practices.

Based on previous literature, ^23, 25^ EA has a broad connotation, and this not only relates to knowledge about the environment but also the attitudes, values, and skills needed to solve environmental issues, which facilitate the ability to carry out responsible civic behaviour. ^22, 31^ Students have not acquired the skills needed to care for the environment, because teachers have not been able to facilitate the environment-based learning process effectively. Therefore, in this study, the researchers used SEA to perform a students’ profile analysis to address environmental problems.

## Methods

Students from middle school that live between urban and rural areas were selected for this study. Researchers’ assumption is based on analysing the profile of students from Senior High School State 1 Selomerto Wonosobo, Central Java Province, to study the EA profile of high school students in Wonosobo Regency. To build environment friendly characteristics among high school students in the era of globalisation, environmental education is crucial. The increasingly sophisticated flow of globalization has eroded pre-existing traditions and cultures because all human life activities are based on technological literacy. ^32^ Thus, the EA profile of high school students will explain the lack of environmental awareness and care among students. Through an analysis of the findings, the criterion variable of each indicator, including those meeting and not meeting the Confirmatory Factor Analysis criteria, can be identified and a fair assessment of the solutions can be achieved.

## Study design and participants

A survey was conducted among students from Senior High School State 1 Selomerto Wonosobo, Central Java Province, Indonesia. The sample was determined based on the purposive sampling technique, meeting the research criteria, that is, the family background of students who lived in the central area of the city who all had similar attributes in terms of cultural and environmental recognition.

The inclusion requirements of this study were as follows.

1) Male and female students who are actively registered as students at Senior High School 1 Selomerto, Central Java Province, Indonesia.
2) Willing to become informants.
3) Physically and mentally healthy.
4) Students’ responses are not influenced by the opinions of teachers, friends, guardians of students or others.
5) Students in class 10-12 only.

A total of 143 students from the entire population in the school filled out the questionnaires after receiving a technical explanation from the researcher. Participation in this survey was voluntary, and no financial reward was offered. All survey procedures and data were guaranteed to be strictly confidential.

Sample size was determined based on the Slovin formula: $n=\frac{N}{1+{\mathrm{Ne}}^{2}}$ , where n is the number of samples; N is the total population ( *N* = 185); and *e* is error tolerance (5%). ^33^ The number of survey data represents the population with a minimum of 126 (131 > 126) samples collected. The total number of students was 143, and 131 gave complete responses. The research sampling distribution is presented in Table 1.

**Table 1.** The research sampling distribution.

Sampling Process	Characteristic	Number of samples	Criteria
Defining population	Senior High School State 1 Selomerto Wonosobo, Central Java Province, Indonesia	185	Non probability with purposive sampling
Developing sampling frame	Meet the inclusion criteria	143	The collected survey questionnaires had 131 complete responses out of a total 143 because 12 responses were incomplete and had to be deleted
Determining Sample Size	Slovin formula’s	Population with a minimum of 126 (131 > 126)	The number of samples 131 have met the minimum requirements for sampling
Selecting Sample	Meet the inclusion criteria and represent the population	131	1) Class 10, 45 responses; class 11, 46; and class 12, 40 2) Demographics of respondents based on gender were 44% male and 56% female. Age range 15-19 years with details of age 15 years, 26%; 16 years, 44%; 17 years, 24%; 18 years, 4%; and 19 years, 2%.

### Data collection

The questionnaire data collection process was adjusted to a 5-point Likert scale, namely: strongly agree, 5; agree, 4; neutral, 3; disagree, 2; and strongly disagree, 1.

The collected survey questionnaires had 131 complete responses out of a total 143 because 12 responses were incomplete and had to be deleted. Twelve samples had missing data, because they do not completely answer all the questions, and the remaining 54 samples were ignored because the sample size was reached.

A questionnaire and a SEA instrument (see *Extended data* ^61^) was used for data collection, consisting of 42 questions about environmental issues faced by the community. The questionnaire was filled in directly by students via the Google Form link ( https://bit.ly/2MXA4HY) within a 2-month research period, from 8 April to 8 June 2020.

The profile of students’ environmental care attitudes was determined based on six EA indicators developed in the SEA instrument; each indicator had three sub-indicators. The assumption of EA is based on the conscious mind to regulate reason, which is a part of the attitude naturally forming social issues. ^6, 21^ This implies not only knowledge of the environment but also the attitudes, values, and skills needed to responsibly solve environmental issues. ^21^ The indicators of students’ EA and the relevance of environmental learning are explained in Table 2.

**Table 2.** Environmental awareness (EA) indicator ^1^.

Indicator	Symbol	Description
Care	EA 1	EA 1a. Care towards environmental damage EA 1b. Care towards dangers to the environment EA 1c. Care towards environmental health
Curiosity	EA 2	EA 2a. Curiosity about how to preserve the environment EA 2b. Explore knowledge about environmental health EA 2c. Find out how to solve the problems related to environmental damage
Critical	EA 3	EA 3a. Having ideas to protect the environment EA 3b. Contributing towards preserving the environment EA 3c. Solving environmental problems
Dependability	EA 4	EA 4a. Reliable for protecting the environment EA 4b. Reliable for preserving the environment EA 4c. Has an excellent attitude towards preserving the environment
Responsibility	EA 5	EA 5a. Response towards the dangers of environmental damage EA 5b. Response towards preservation of environmental health EA 5c. Aware of the dangers of environmental damage
Local Wisdom	EA 6	EA 6a. Preserves the local potential of the environment EA 6b. Aware of local potentials EA 6c. Protects local wisdom as the way to show respect and empathy

## Data analysis

Quantitative descriptive data analysis was used to assess the students’ responses to the EA questionnaire. ^34^ A t-test using LISREL 8.8 Second-Order CFA was used to check the measurement results to ensure that there are no offending estimates (the values that exceed accepted limits) on the variables. The EA indicators observed in each latent variable fulfilled the analysis requirements by linking and matching sub-indicators and indicator, one indicator with another, and combining the criteria components on EA indicators into one SEA model.

LISREL 8.8 Second-Order CFA confirms variables based on factor analysis, so that students’ profiles are validly and reliably measured. The LISREL method was applied, and the results showed that output of solution standard in identifying the student’s EA profile problems are based on t-test scores, analyse expected changes to provide solutions, and provide recommendations based on results of the analysis. The LISREL 8.8 Second-Order CFA application identifies the relationship between complex environmental care attitude variables and the sub-indicators meeting statistical requirements. LISREL 8.8 Second-Order CFA analysis is very sensitive for ambiguous data and predicts every indicator of the question.

The dependence of one indicator’s data on other indicators may result in a mismatch, so the instrument was been validated by an expert in SEA instruments. The criteria for the validator of the SEA instrument are one environmental expert lecturer and one evaluation tool lecturer. The purpose of validation provides an assessment of the feasibility of the SEA instrument, and whether or not it can be used in the data collection process.

The revised SEA instrument validation are as follows.

• The instrument has been tested to find out content validation and construct validation on students' environmental awareness questionnaires and the results meet the valid criteria.
• Each question has presented social issues contextually to answer each question so that research focuses on environmental studies.
• The questionnaire grid has represented each indicator of the measured variable.

The instrument was been revised based on the validator’s suggestions with the final decision that the SEA instrument is suitable for measuring students’ EA profiles.

LISREL software is proprietary software; a freely available alternative software that can be used to perform the same analysis is lavaan: https://cran.r-project.org/web/packages/lavaan/.

*Ethical considerations*

The Universitas Sebelas Maret Surakarta gave permission for the study to be conducted on 7 ^th^ January 2020 (letter number 40/UN27.02.9.2/DP/2020). This research was also approved by the Senior High School State 1 Selomerto, Wonosobo, Central Java Province, Indonesia (letter number 800/208/2020; dated 8 April 2020).

Written consent to participate was obtained from students, student guardians, and the principals of participating schools. If consent from student guardians was not obtained, the student was not allowed to participate. Respondents provided consent without any coercion from anyone. All forms of data obtained will remain confidential to protect the rights and privacy of the respondents.

## Results

**Figure 1.**
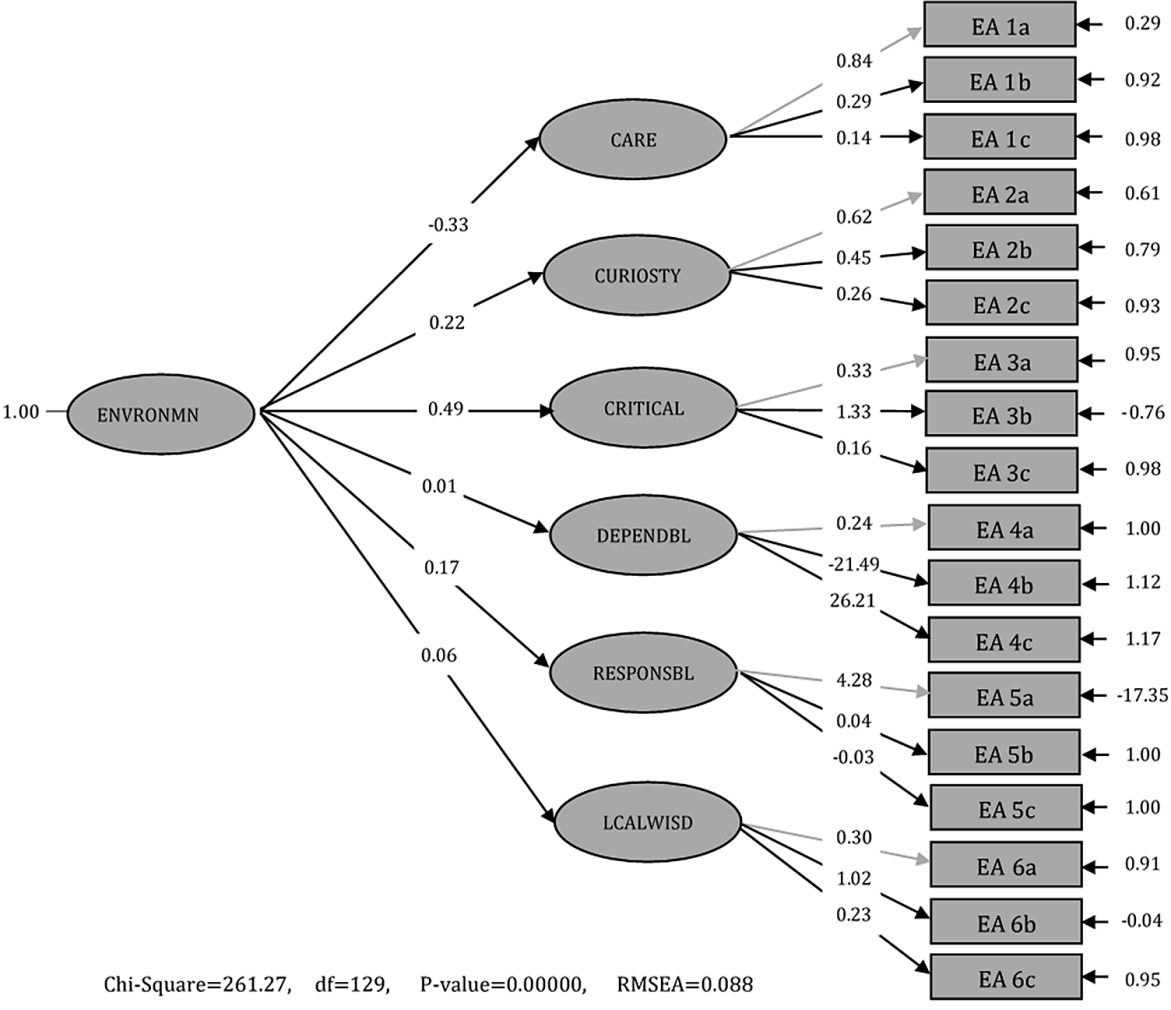
Standard solution second-order confirmatory factor analysis results.

The number of potential respondents who met the sample requirements was 143, but in the research process there were 12 students who did not give a complete response. Therefore, the data that were analysed were 131 respondents who gave complete answers. The results for the respondents’ answers to the questionnaire and observation of students’ EA profile ^35^ are shown in Figure 1.

Based on Figure 1, the observation variable (indicator) has a larger value of convergent validity (factor loading) than 0.5; the fit model in P-value is 0.25>0.05; Root Mean Square Error of Approximation (RMSEA) is 0.088; and Normed Fit Index (NFI) is 0.67. The value of the loading factor in indicators A1, A3, and A5 <0.5 and for indicators A2, A4 and A6 >0.5. The t-value is <1.96, so it fulfils the significance criteria. The results show that for indicators Care (EA I), Curiosity (EA 2), Critical (EA 3), Dependability (EA 4), Responsibility (EA 5), and Local wisdom (EA 6), the SEA instrument is valid. Therefore, the SEA instrument is valid and reliable to measure senior high school students’ EA. The development of the SEA instrument can be used as a standard measuring instrument to determine students’ EA through physical learning about temperature, heat, and global warming. The results of the t-value are presented in Table 3.

**Table 3.** T-value second-order confirmatory factor analysis results.

Indicator	Symbol	Indicator	T-value
Care	EA 1	EA 1a EA 1b EA 1c	0.46 6.82 8.02
Curiosity	EA 2	EA 2a EA 2b EA 2c	2.36 4.77 7.46
Critical	EA 3	EA 3a EA 3b EA 3c	7.84 0.64 8.14
Dependability	EA 4	EA 4a EA 4b EA 4c	8.15 6.06 5.09
Responsibility	EA 5	EA 5a EA 5b EA 5c	116,31 8.17 8.16
Local Wisdom	EA 6	EA 6a EA 6b EA 6c	5.96 0.03 7.29

Table 3 shows that the Care sub-indicator (EA 1a) does not fulfil the significance criteria 0.46 or less than 1.96 (0.46<1.96). The sentence needs to be revised from ‘care the environment’ to ‘Care towards environmental damage’. During testing of the questionnaire, there were sentences in the Care sub-indicator that were unclear. Therefore, it was necessary to revise the sentence about environmental damage by providing an example of an unpleasant smell in the Sikidang Crater area – this has been previously shown to worsen the health of society and requires global environmental care.

The Critical sub-indicator (EA 3b) does not fulfil the loading factor; it only amounts to 0.64. ‘Contributing towards preserving the environment’ as a form of critical thinking on environmental problems. The example given on the questionnaire was *PranotoMongso,* which has disappeared today because farmers in Dieng use a modern system of agriculture, using plastics on agricultural land ( Figure 3). The farmers do not realise that the use of chemical fertilisers and modern farming tools is not environment friendly, and can harm the fertility of the land and destroy the ecosystem.

**Figure 2.**
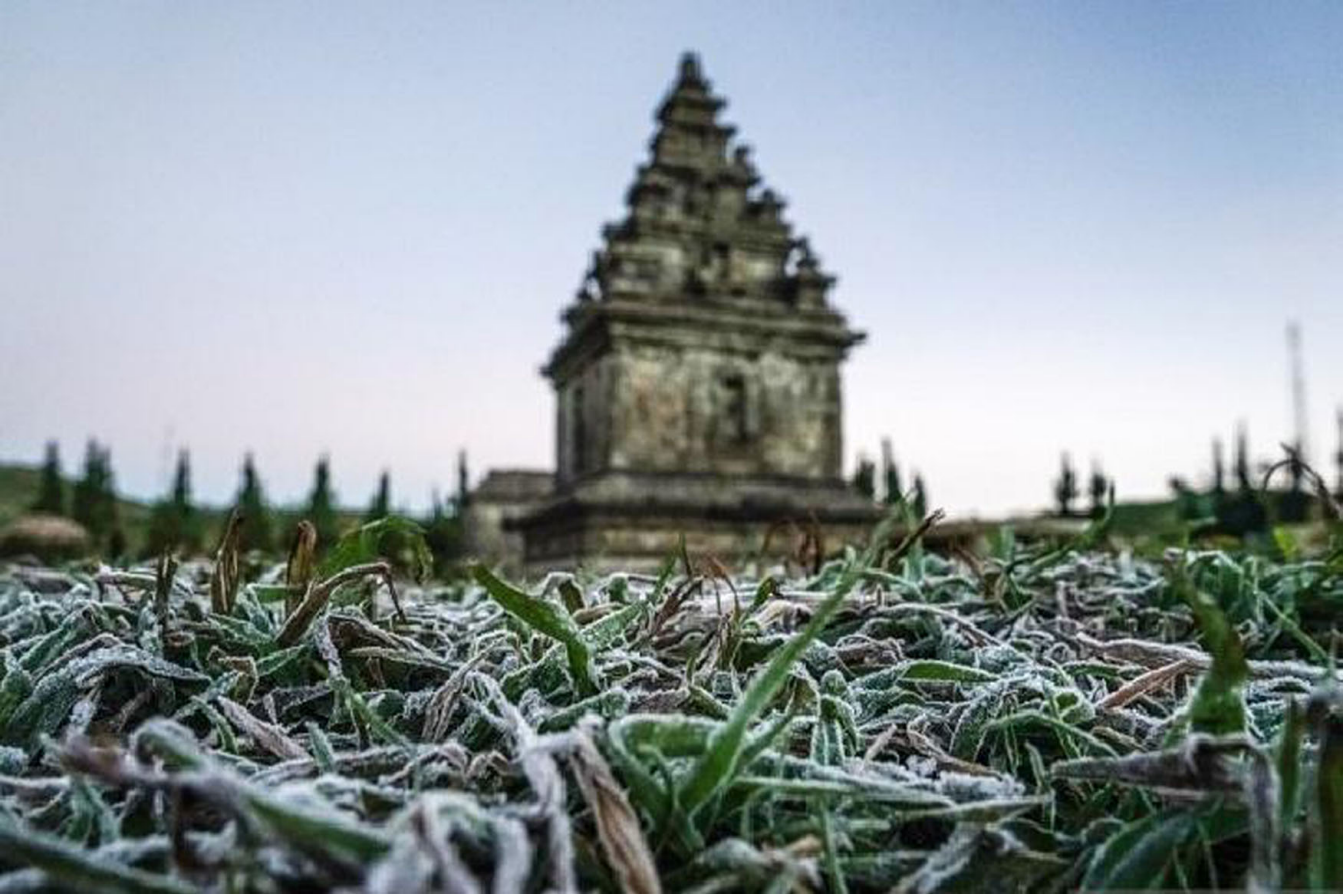
Weather anomaly ‘ *Embun Upas*’.

The Local Wisdom sub indicator (EA 6b) did not fulfil the factor loading 0,03. Therefore, re-evaluation is needed to obtain a good profile of EA. For the ‘aware of local potential’ indicator, the capability to understand the local potential and conserve local potential that does not match the indicator criteria. The example given on the questionnaire was the dew phenomenon or ‘Embun Upas’, as shown in Figure 2 (discussed further below). Due to the cold at night and freezing dew in several plateaus in Indonesia, like in Dieng, is caused by meteorological conditions and dry season. Java is now at the top of the dry season, which can be observed from the fact that several mountains have 0°C. This is because the air molecules in mountainous areas are more tenuous than the lowlands; they experience rapid cooling, especially when the weather is clear and not covered by clouds or rain, and water vapour in the air at night leads to condensation, sticking to leaves or grass and immediately freezing because of the temperatures.

**Figure 3.**
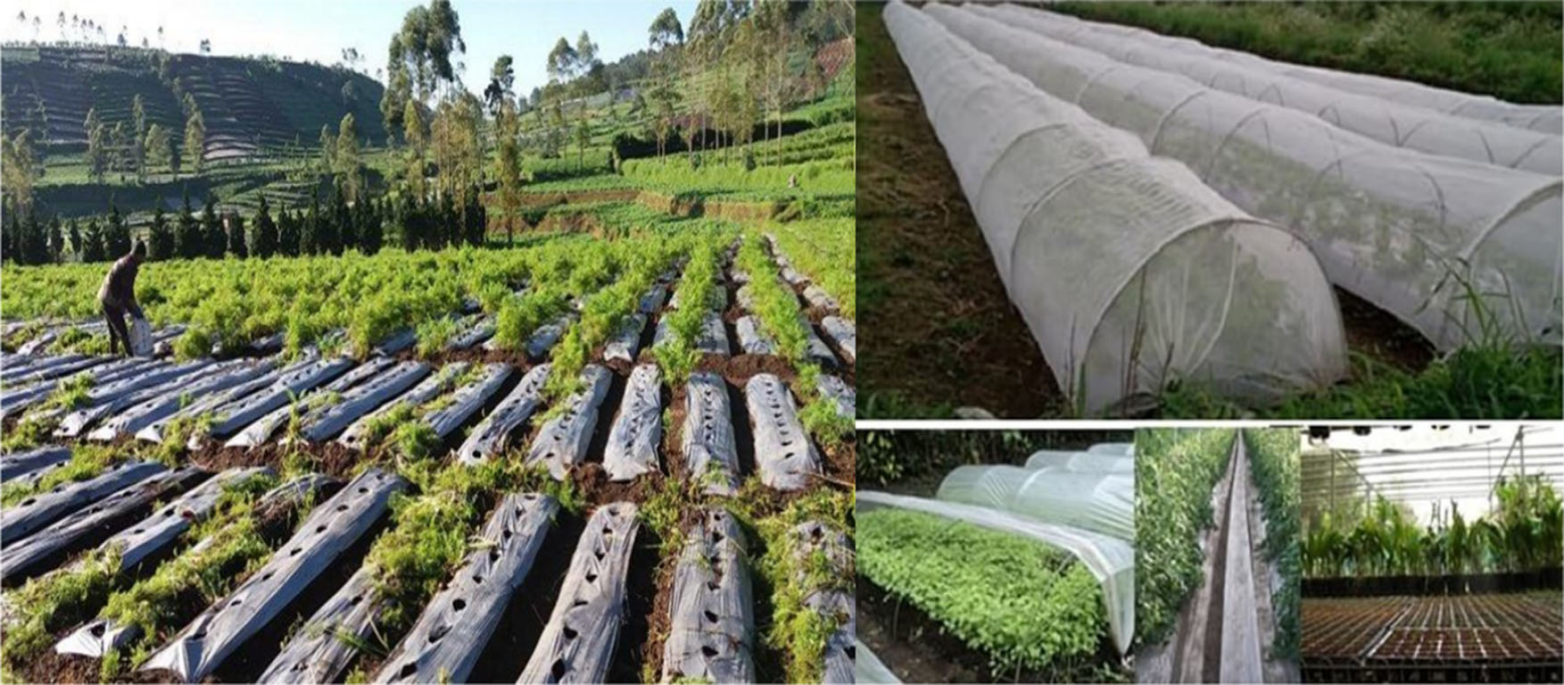
The use of plastic in agriculture in Dieng Plateau.

Three profiles are not qualified with a t-value of less than 1.96 from six indicators with three sub-indicators. There are 18 sub-indicators of EA with t-values in the highest responsibility indicators (EA 5a). It is a ‘Response towards the dangers of environmental damage’. The students agree that the environment is a part of their lives and must be protected and preserved. Science subjects and culture must be included in learning at school.

The care indicator (EA 1a) ‘care towards environmental damage’, critical indicator (EA 3b) ‘Contributing towards preserving the environment’, and local wisdom sub-indicator (EA 6b) to ‘Aware of local potentials’ are not fulfilled. The lack of EA and understanding about the potential make students unaware of the need to protect the environment, even though they know about the dangers of environmental damage. This means that students must have environmental care attitudes to initiate a reciprocation between students and nature. The profile of school students’ EA is good. However, it needs to be improved through the result of the expected change second-order CFA analysis. Furthermore, analysis of the suitability of the SEA model used in measuring students’ EA profiles is presented in Table 4.

**Table 4.** Expected change second-order confirmatory factor analysis results.

Indicator	Symbol	Indicator	Value	Expected change
Care	EA 1	EA 1a EA 1b EA 1c	38.88 0.00 0.24	
Curiosity	EA 2	EA 2a EA 2b EA 2c	0.00 0.00 0.00	0.28
Critical	EA 3	EA 3a EA 3b EA 3c	0.00 0.00 0.00	0.25
Dependability	EA 4	EA 4a EA 4b EA 4c	0.00 0.00 0.00	0.37 0.33
Responsibility	EA 5	EA 5a EA 5b EA 5c	0.00 0.00 0.00	0.27
Local Wisdom	EA 6	EA 6a EA 6b EA 6c	0.39 0.00 0.00	

The potential of the EA instrument is expected to be changed and revised in terms of measuring every good indicator from changes to the sentence on the questionnaire to make it clearer so that the research results are valid.

**Figure 4.**
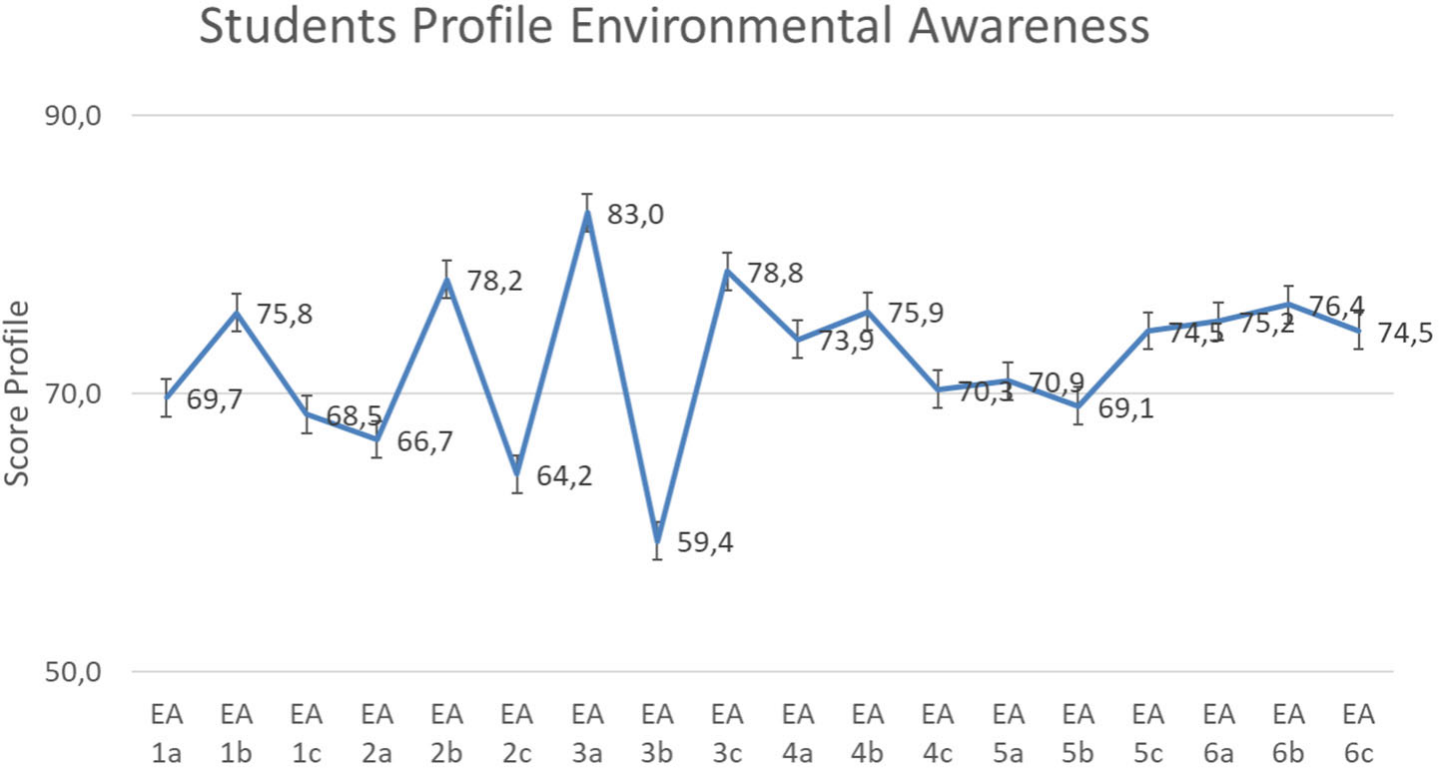
Environmental awareness sub-indicator profile.

Based on the result of descriptive counting, for environmental care data, the scores ranged from 59.4 sub-indicator ‘contributes towards environmental sustainability’ to 83.0 in the sub-indicator ‘know about preserving the environment’ as shown in Figure 4.

**Figure 5.**
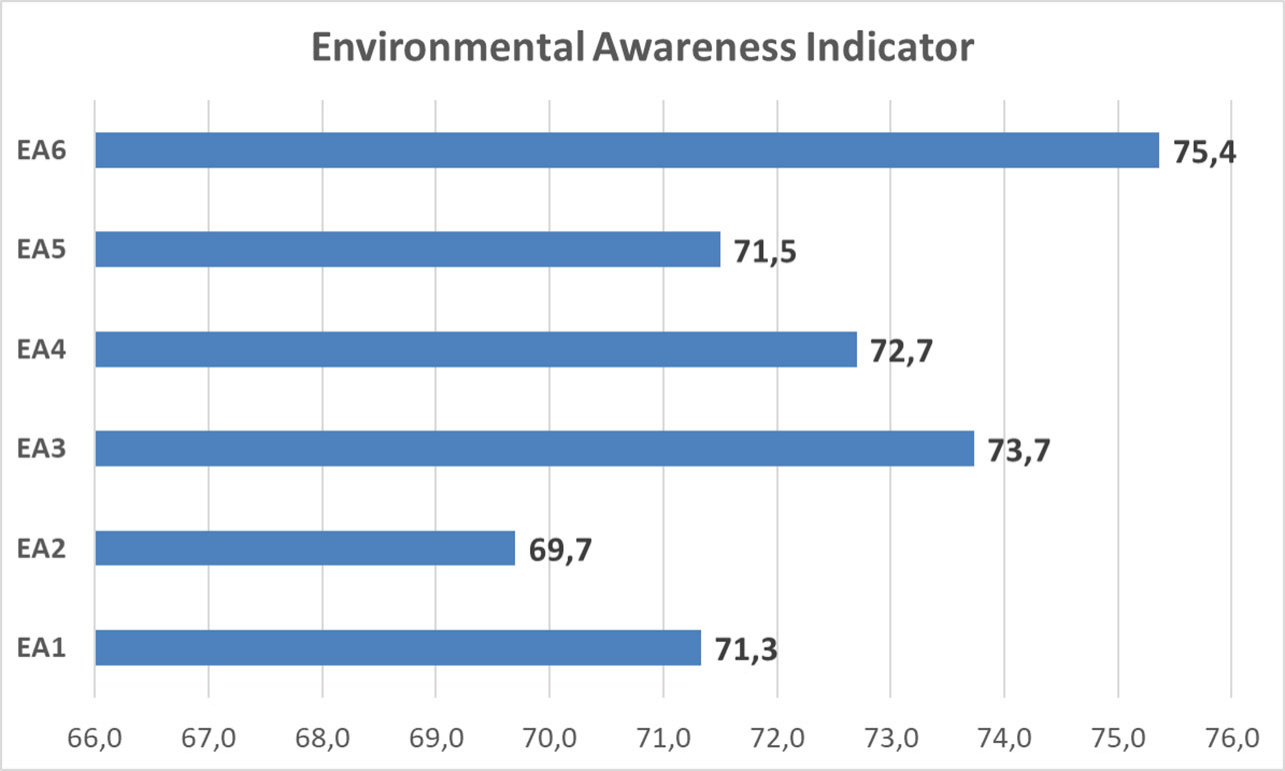
Environmental awareness indicator profile.

Based on Figure 4, the environmental care of students show the value 72.4 on an average in ‘enough category’, with the highest indicator score for ‘local wisdom’ (75.4). Meanwhile, ‘curiosity’ has the lowest score (69.7). This is shown in Figure 5.

The value of the dominant tendency profile of environmental care is in the sufficient category with a score of 72.4 on average. Therefore, further analysis is required to identify sub-indicators that fulfil or do not fulfil the criteria determined in the SEA instrument.

## Discussion

The results of confirmatory factor analysis showed an evaluation of the goodness of fit, t-value, and the expected change in identifying the EA profile of high school students. The change in the EA indicator ( Table 4) is a crucial factor for creating a strategy to deal with environmental issues in society. The context-based learning benefits students and enhances their awareness regarding cultural preservation. ^38^

The importance of strategy in an environment-based teaching model, while respecting local culture, is an ethnoscience approach. Ethnoscience is used as a reference to equip students with knowledge and character so that they respect their culture ( Figure 2 and Figure 3). Ethnoscience learning is a means of self-development, increasing awareness to participate in preserving the environment and cultural traditions. This component has been shown in ethnoscience-based learning to improve students’ EA. ^1, 6^ Unfortunately, technology is becoming increasingly advanced and literacy has failed to improve social behaviour. Besides the effect on the education system that utilizes formal science from indigenous knowledge in the form of customs, local wisdom, and cultural traditions as learning resources, high school students do not focus on their interactions with the environment. ^35, 36^ The primary source of knowledge that can be effectively obtained is through direct interaction between students and nature, as opposed to studying concepts in classrooms.

Ethnography-based teaching will improve student’s skills in science, respect their achievements, and improve their skills to implement their knowledge. Through this teaching method, students’ understanding of science in a cultural context will be improved since they directly learn about the environment. Therefore, they will have a curiosity for, and give attention to, customs and culture. Thus, teaching improves their creativity. ^41^

Socioscientific issues (SSI) based on SEA is a teaching strategy that explains science subjects in terms of social problems involving moral or attitude components. ^39, 40^ SSI is a common conceptual or procedural problem related to science and has a rational solution, influenced by social aspects, such as culture, politics, economy, and ethics. ^39, 41^ The involvement of social aspects in SSI provides an opportunity to create conflict between scientific and social perspectives. Teaching can help develop moral reasoning and reflective assessment skills in terms of problem-solving. ^39, 40, 42^

Environmental education impacts society by enhancing students’ awareness of the environment. ^7, 43, 44^ To integrate the ethnography approach as a source of science teaching in high schools using the SSI strategy to empower EA related to environmental damage uses a strategic environmental study ^48^ in terms of learning. ^49^

A contextual aspect is required in environmental learning, since the scope of the problem is related to daily life, which pertains not only to knowledge but also to attitudes and skills to solve environmental issues. ^50^ One of the potential learning strategies that can be implemented is SSI.

Culture, as a part of the social life for different generations, also requires attention. Based on the purpose of learning to enhance students’ knowledge and understanding about life through values and attitude, ^51^ the transition of students into future physics teachers requires excellent preparation.

Skills can be gained from an ethnoscience learning model. Ethnoscience improves students’ knowledge and develops local wisdom and uses formal physics teaching as a source of learning in universities, where ethnoscience is a form of experience and culture. ^41^ Further, it can improve students’ capabilities to implement their scientific knowledge.

The quality of science learning that reflects the social context as an environmental problem is indicated by the existence of authentic topics or issues that develop in society. The topic is relevant if students’ decisions influence their present and future lives. The science learning curriculum is reflected to point them in the direction of the impact. Moreover, SSI evaluation makes solving problems possible from various perspectives. An open discussion in specific forums helps students to understand social problems that develop, except for religious or ethnic issues.

Further, analysing science and technology as a tool for teaching, raises an informal logical question, which relates to scientific fact. It is explained either explicitly or implicitly as an argumentation subject. ^52, 53^ Therefore, the environmental-based science teaching process is more valuable than pure experimental activities. ^38, 54^ To design teaching based on socio-cultural problems, teachers need scientific knowledge and consideration of the social aspect. In addition, teachers also need to realise that in terms of teaching, there are uncertainties in the class. They must realise that teaching is not the only way to assert authority. ^55^

Teaching based on ethnoscience improves students’ skills, which are required to process science, appreciate, and protect the environment. The student also learns to use their scientific knowledge. ^40^ Through this method, every student will understand the concepts of science in cultural context, since they learn directly from the environment. ^56^ Therefore, there is a student’s curiosity and concern for the customs and culture that are learned to shape students’ environmental care profile, using strategic environmental studies, facilitated by modifying the ethnoscience approach to explore local potential, customs, and culture, which is crucial. Meanwhile, SSI is used to help them gain the knowledge to identify current issues in society and provide a positive response, protect the environment from danger, and understand and appreciate the local wisdom.

EA is shown based on awareness, protection, and preservation, especially unfulfilled local potential. ^4, 5, 21^ This study contributes to a critical analysis of EA profiles through LISREL Second-order CFA by revealing latent variables or indicators ( Figure 1 and Tables 3-4) that affect other indicators. The factor analysis indicates that the students’ environmental care attitude is not only limited to caring, appreciating, and having good environmental ethics, but also includes making a real contribution and finding solutions to environmental problems that are integrated into the learning process.

There are rapid changes in the social behaviour, and the roles of humans are increasingly being replaced by sophisticated digitalization systems. ^45, 54, 55^ Students are becoming increasingly indifferent about preservation of their surrounding environment. ^8, 9^ To avoid this, incorporating students’ direct experience acts as a useful learning resource, compared with learning where students only experience abstract concepts. Therefore, concerns about advances in technological literacy and culture, leading to an abandonment of environment in society can be resolved. Environmental literacy and competence are actively dedicated towards solving problems in human–environment interactions in an ecological and humanist way. The higher purpose is to balance the quality of life and the quality of the environment, with non-formal training in improving environmental literacy. ^58^

Filho ^59^ explains that the perspective of life is the basis for moral formation and has a very complicated relationship with EA, environmental knowledge, and human behaviour. Simultaneously, our study holistically analyses the profile of students’ EA by considering policies, plans, and environmentally sustainable programs. Furthermore, according to Murniawaty ^5^ and Widodo, ^60^ knowledge and commitment are needed to realise environmental protection and awareness, but they have not revealed how practical solutions can be used to implement them.

Based on the results of our analysis, the limitation of the study lies in the student bias data, namely the results are the same between the answers to ‘positive’ statements and ‘negative’ statements. It is assumed that there are irresponsible student answers. The results of this data cause several indicators and sub indicators to be not fulfilled ( Figures 4 and 5). Furthermore, individually interviewing every student was not possible due to limited time and research costs. The study is significant in the sense that it illustrates how an increase in students’ EA profile can be determined through the interpretation of the LISREL 8.8 second-order CFA analysis, influenced by the relationship between EA indicators and sub-indicators with standardised SEA. Research recommendations in the form of measuring the EA profile of students using SEA are appropriate for considering the implementation of environmental education, integrated with ethnoscience and issues that develop in society. These are attained while realising the character of students who care for and respect the environment.

## Conclusions

The profile of environmental awareness (EA) of high school students is in the sufficient category with a Care indicator (EA 1a). ‘Care towards environmental damage’, Critical (EA 3b), ‘Contributing towards preserving the environment’, and Local wisdom (EA 6b) to ‘Aware of local potentials’ are not fulfilled. Their lack of EA and understanding of potential fails to contribute to the environment. Their responses to environmental damage are good. It is highly necessary to implement SEA, which is modified with the ethnoscience approach and environmental issues strategy to build the character of students so that they develop an awareness of their environment. Further research should be conducted to study its contributions towards EA students who not only appreciate regional potential and ethics in ethnoscience studies but can also find real solutions to environmental problems with the socioscientific issues strategy. Furthermore, the synergy between teachers, students, and policymakers in the implementation of environment-based education is crucial for realising character through student EA.

## Data availability

### Underlying data

Figshare: Environmental Awareness Questionnaire Score through SEA Assessment. https://doi.org/10.6084/m9.figshare.13977707.v1. ^34^

This project contains the raw data of student’s response.

Figshare: Environmental Awareness Result. Environmental Awareness Result. https://doi.org/10.6084/m9.figshare.14175563.v1. ^35^

This project contains data that has analysed the scores obtained by each EA indicator, the actual score and the percentage.

### Extended data

Figshare: Questionnaire for High School Student Environmental Awareness Profiles, https://doi.org/10.6084/m9.figshare.14254106. ^61^

Data are available under the terms of the Creative Commons Zero “No rights reserved” data waiver (CC0 1.0 Public domain dedication).
