# Supplementary material for: Analysing students’ environmental awareness profile using strategic environmental assessment
Source: F1000Res. 2021 Jul 21;10:305. Originally published 2021 Apr 20. [Version 2] doi: 10.12688/f1000research.51523.2 (PMC8956850; doi:10.12688/f1000research.51523.2)
Supplement: Peer review history

# Peer review: Reviewer response for version 2

**DOI:** 10.5256/f1000research.58623.r126892

**Published:** 2022-04-06

**Authors:** Montserrat Franquesa-Soler

**Affiliations:** 1 Universidad Popular Autónoma del Estado de Puebla (UPAEP), Puebla, Mexico

In general, the Introduction lacks organization. From the more general and international information to more concrete and national (regional/local) information. The last paragraph should provide a strong justification of the study, and methods. In the end, clearly state the Main Goal and Particular Goals.

**Details of Introduction:**

 Some statements would need citations (second sentence and third sentences). For the first sentence, I suggest including the Socioecosystem paradigm/approach in the Introduction briefly, here is an article as a reference ^1^. The three first sentences are not well connected. The degradation of environmental conditions can worsen the development of science education - how? I think the contents of science education need to be updated and focused and these new dynamics that the Planet is undergoing (e.g., environmental impact and how through science this situation can be tackled). 

*"Environmental issues remain under-studied and under-utilized in science learning"* - I do not agree with the first part of the sentence, because there is indeed research focused on environmental issues in science learning. I suggest authors double-check the literature and incorporate these findings. E.g., Litteldyke, M. (1997), Littledyke, M. (2008) ^2^, Zhongming, Z., Linong, L., Xiaona, Y., Wangqiang, Z., & Wei, L. (2021). 

*"Environmental care, as it has been taught in school, refers to definitions, terms, concepts, and knowledge about the natural environment" - *according to who? citation missing.

**Details of methods:**

 There are some statements that belong to the Introduction. For E.g. *"To build environment-friendly characteristics among high school students in the era of globalization, environmental education is crucial. The increasingly sophisticated flow of globalization has eroded pre-existing traditions and cultures because all human life activities are based on technological literacy".*

Study design: The percentage of masculine/feminine/age groups is missing.

**Details of Discussion and Conclusion:**

 I suggest including Participatory Action Research ^3^ topic to enrich some parts of the recommendations and next steps in this research.

Is the work clearly and accurately presented and does it cite the current literature?

Partly

If applicable, is the statistical analysis and its interpretation appropriate?

I cannot comment. A qualified statistician is required.

Are all the source data underlying the results available to ensure full reproducibility?

Yes

Is the study design appropriate and is the work technically sound?

Yes

Are the conclusions drawn adequately supported by the results?

Yes

Are sufficient details of methods and analysis provided to allow replication by others?

Partly

Reviewer Expertise:

Environmental Education; Participatory Action Research, Sustainability in Higher Education; Environmental perceptions; Primate Conservation

I confirm that I have read this submission and believe that I have an appropriate level of expertise to confirm that it is of an acceptable scientific standard, however I have significant reservations, as outlined above.

## References

[1] : Socio–Ecosystemic Sustainability. *Sustainability* .2019;11(12) : 10.3390/su11123354 10.3390/su11123354

[2] : Science education for environmental awareness: approaches to integrating cognitive and affective domains. *Environmental Education Research* .2008;14(1) : 10.1080/13504620701843301 1-17 10.1080/13504620701843301

[3] : Using participatory action research to operationalize critical systems thinking in social-ecological systems. *Ecology and Society* .2020;25(1) : 10.5751/ES-11369-250116 10.5751/ES-11369-250116

# Peer review: Reviewer response for version 2

**DOI:** 10.5256/f1000research.58623.r126893

**Published:** 2022-03-25

**Authors:** Ebtsam Aly Abou Hashish

**Affiliations:** 1 Nursing Administration Department, Faculty of Nursing, Alexandria University, Alexandria, Egypt

Thanks for the opportunity to review this interesting paper. Generally, it is a well-written paper, and it gets better after review.

 Consider the few following comments:

**LISREL:**

- Write the complete name for the first time.
- Write the type of sample (purposive).

 
**Methods:**

- A descriptive survey design.
- Non-test questionnaires - What do you mean by this?
- *"Quantitative descriptive data analysis on EA indicators (Care, Curiosity, Critical, Dependability, Responsibility, and Local Wisdom)"* - Add 'was done' to the end of the sentence.

**Tools:**

- Please organize your tools in the following format:
  Write the name of the questionnaire, developer and year, how many dimensions and how many items for each dimension, the scale and the score, reliability alpha.
- Add the demographic form and include items for your tool.
- Where is the validity and reliability of the tool used?
- Did you conduct a pre-test or pilot study for your tool?
- Again, for data analysis, LISREL: write completely for the first time, then use the abbreviation.

**Conclusion:**

- Include a clear strategy for increasing EA.

Is the work clearly and accurately presented and does it cite the current literature?

Yes

If applicable, is the statistical analysis and its interpretation appropriate?

Yes

Are all the source data underlying the results available to ensure full reproducibility?

Yes

Is the study design appropriate and is the work technically sound?

Yes

Are the conclusions drawn adequately supported by the results?

Yes

Are sufficient details of methods and analysis provided to allow replication by others?

Yes

Reviewer Expertise:

Nursing research - education, leadership and management - education- ethics

I confirm that I have read this submission and believe that I have an appropriate level of expertise to confirm that it is of an acceptable scientific standard.

# Peer review: Reviewer response for version 2

**DOI:** 10.5256/f1000research.58623.r90017

**Published:** 2021-08-02

**Authors:** Sarwi Sarwi

**Affiliations:** 1 Faculty of Mathematics and Natural Sciences, Universitas Negeri Semarang, Central Java, Indonesia

The revision is in accordance with everything suggested by the reviewer. After I looked at he published revision of the article it, I gave my approval to be indexed.

Is the work clearly and accurately presented and does it cite the current literature?

Yes

If applicable, is the statistical analysis and its interpretation appropriate?

Partly

Are all the source data underlying the results available to ensure full reproducibility?

Yes

Is the study design appropriate and is the work technically sound?

Yes

Are the conclusions drawn adequately supported by the results?

Yes

Are sufficient details of methods and analysis provided to allow replication by others?

Yes

Reviewer Expertise:

The exact sciences, environmental sciences, innovative science learning

I confirm that I have read this submission and believe that I have an appropriate level of expertise to confirm that it is of an acceptable scientific standard.

# Peer review: Reviewer response for version 1

**DOI:** 10.5256/f1000research.54703.r87791

**Published:** 2021-07-01

**Authors:** Latifah Abd Manaf

**Affiliations:** 1 Department of Environment, Faculty of Forestry and Environment, Universiti Putra Malaysia, Serdang, Malaysia

Some changes:

- Conflict statement in the abstract (part results and conclusion) regarding students’ awareness.
- The last paragraph in the introduction section is a method part, this can be removed.
- Discussion about the students as the respondents should be in the method part. Rearrange.
- Add info on the sample distribution in the table and add more discussion on the sampling method for the respondent selection.
- Add more discussion on the instrument validation and pilot study.
- Questions for each indicator were only 3? Is it valid to evaluate the SEA?
- Information on the demographic background of respondents must be in the Table and discuss.
- It’s better to discuss the results directly by supporting them with references, instead of putting them under a separate section.
- Figure 4 and 5 is a result part.
- The conclusion can be improved.

Is the work clearly and accurately presented and does it cite the current literature?

Yes

If applicable, is the statistical analysis and its interpretation appropriate?

Yes

Are all the source data underlying the results available to ensure full reproducibility?

Yes

Is the study design appropriate and is the work technically sound?

Yes

Are the conclusions drawn adequately supported by the results?

Partly

Are sufficient details of methods and analysis provided to allow replication by others?

No

Reviewer Expertise:

NA

I confirm that I have read this submission and believe that I have an appropriate level of expertise to confirm that it is of an acceptable scientific standard, however I have significant reservations, as outlined above.

# Response

**Published:** 2021-07-10

**Authors:** Ahmad Khoiri

Q.1: Conflict statement in the abstract (part results and conclusion) regarding students’ awareness.

 A.1: The EA profile of high school students was categorised sufficiently. Conclusions: Students’ lack of awareness of the environment and understanding of their regional potential fails to contribute towards creating a sustainable environment. 

 Q.2: The last paragraph in the introduction section is a method part, this can be removed.

 A.2: The last paragraph has been moved to the methods section.

 Q.3: Discussion about the students as the respondents should be in the method part. Rearrange.

 A.3: Respondents have been described in the Study design and participants section.

 Q.4: Add info on the sample distribution in the table and add more discussion on the sampling method for the respondent selection.

 A.4: Table 1 has been added regarding the research sampling distribution.

 Q.5: Add more discussion on the instrument validation and pilot study.

 A.5: Added, The results of the revised SEA instrument validation are as follows.

- The instrument has been tested to find out content validation and construct validation on students' environmental awareness questionnaires and the results meet the valid criteria.
- Each question has presented social issues contextually to answer each question so that research focuses on environmental studies.
- The questionnaire grid has represented each indicator of the measured variable.

Q.6: Questions for each indicator were only 3? Is it valid to evaluate the SEA?

 A.6: A valid criterion instrument that has been tested based on expert judgment and instrument testing.

 Q.7: Information on the demographic background of respondents must be in the Table and discuss.

 A.7: Added to table 1 about demographics of respondents based on gender were 44% male and 56% female. Age range 15-19 years with details of age 15 years, 26%; 16 years, 44%; 17 years, 24%; 18 years, 4%; and 19 years, 2%.

 Q.8: It’s better to discuss the results directly by supporting them with references, instead of putting them under a separate section.

 A.8: the article has been adapted to the journal guidelines for separate results and discussion.

 Q.9: Figures 4 and 5 is the result part.

 A.9: Figures 4 and 5 have been presented in the results section.

 Q.10: The conclusion can be improved.

 A.10: The conclusion has answered the research objectives and provided recommendations, added the criteria for the category of student environmental awareness are sufficient category.

# Peer review: Reviewer response for version 1

**DOI:** 10.5256/f1000research.54703.r83578

**Published:** 2021-05-10

**Authors:** Sarwi Sarwi

**Affiliations:** 1 Faculty of Mathematics and Natural Sciences, Universitas Negeri Semarang, Central Java, Indonesia

Abstract, please add: Purpose is expressed in an operational sentence to reveal the topic referred to the title of the manuscript. Operational disclosure is required because it affects the adequacy of the required data.

 ​​​​​​Research discussing the importance of environmental issues associated with high school student attitudes is an interesting topic in education. The writing uses relevant and > 90% up-to-date article citations, according to the topics discussed. Please add: The theoretical framework that supports research is built so that the direction of the research is clear so that problems can be resolved.

 The research design is appropriate, but qualitative data are still needed regarding the responses and results of in-depth interviews from respondents to answer research problems. The results of the research have good academic achievement, especially for high school and equivalent education levels.

 Students from middle school that live between urban and rural areas were selected for this study (10 ^th ^paragraph). Writing of that sentence in the text by the author is unclear, and even tends to have opposite meanings.

 Methods: The number of public high schools in Wonosobo Regency which are located in downtown or urban areas is at least 4 schools. Based on this data, it is necessary to refine or reconsider the subject or source of research data.

 Statistical analysis and interpretation can be applied in other institutions provided they have the same characteristics. However, it is still necessary to discuss aspects of attitude EA with low scores, why?

 All data sources are available and can be used to ensure full reproducibility. There is a review that the data on the figure illustration (Figure 2 and Figure 3) is too large so that it is needs revision.

 The conclusions drawn are supported by the results of data processing. Data processing requirements are adjusted to written objectives in an operational and measurable manner.

 References are used both as a guide for the path of ideas (state of the arts) and discussion. Reference is up to date and applicable to the problem.

Is the work clearly and accurately presented and does it cite the current literature?

Yes

If applicable, is the statistical analysis and its interpretation appropriate?

Partly

Are all the source data underlying the results available to ensure full reproducibility?

Yes

Is the study design appropriate and is the work technically sound?

Yes

Are the conclusions drawn adequately supported by the results?

Yes

Are sufficient details of methods and analysis provided to allow replication by others?

Yes

Reviewer Expertise:

The exact sciences, environmental sciences, innovative science learning

I confirm that I have read this submission and believe that I have an appropriate level of expertise to confirm that it is of an acceptable scientific standard.

# Response

**Published:** 2021-05-17

**Authors:** Ahmad Khoiri

**Q.1: **Abstract, please add: Purpose is expressed in an operational sentence to reveal the topic referred to the title of the manuscript. Operational disclosure is required because it affects the adequacy of the required data.

**A.1**: I have added, this study aimed to analyses students’ EA profile of which consisted of six indicators, namely Care (EA 1), Curiosity (EA 2), Critical (EA 3), Dependability (EA 4), Responsibility (EA 5), and Local Wisdom (EA 6).

**Q.2**: Research discussing the importance of environmental issues associated with high school student attitudes is an interesting topic in education. The writing uses relevant and > 90% up-to-date article citations, according to the topics discussed. Please add: The theoretical framework that supports research is built so that the direction of the research is clear so that problems can be resolved.

**A.2**: The theoretical framework has been described in the 4th, 5th, and 9 ^th^ paragraphs.

**Q.3**: The research design is appropriate, but qualitative data are still needed regarding the responses and results of in-depth interviews from respondents to answer research problems. The results of the research have good academic achievement, especially for high school and equivalent education levels.

**A.3**: Qualitative data has been obtained based on respondents' answers through the collected survey questionnaires had 131 complete responses out of a total of 143 because 12 responses were incomplete and had to be deleted. Twelve samples had missing data because they do not completely answer all the questions, and the remaining 54 samples were ignored because the sample size was reached. A questionnaire and a SEA instrument were used for data collection, consisting of 42 questions about environmental issues faced by the community.

**Q.4: **Students from middle school that live between urban and rural areas were selected for this study (10 ^th^ paragraph). Writing of that sentence in the text by the author is unclear, and even tends to have opposite meanings.

**A.4: **that is, the research location is in the middle school (between urban and rural areas). Purposive sampling determination for the family background of students who lived in the central area of the city who all had similar attributes in terms of cultural and environmental recognition. 

**Q.5: **Methods: The number of public high schools in the Wonosobo Regency which are located in downtown or urban areas is at least 4 schools. Based on this data, it is necessary to refine or reconsider the subject or source of research data.

**A.5: **Purposive sampling determination for the family background of students who lived in the central area of the city who all had similar attributes in terms of cultural and environmental recognition. 

**Q.6: **Statistical analysis and interpretation can be applied in other institutions provided they have the same characteristics. However, it is still necessary to discuss aspects of attitude EA with low scores, why?.

**A.6: **EA indicators that score low and do not meet the good fit criteria are presented in Table 2 are (1) Care sub-indicator (EA 1a, 0.46 <1.96); (2) The Critical sub-indicator (EA 3b, 0.64<1.96); (3) The Local Wisdom sub-indicator (EA 6b, 0.03<1.96). Lisrel recommends creating error covariance between indicators and sub-indicators. If this procedure is performed, our new model will experience a decrease in the chi-square value, which of course makes the model better. Each item has a loading factor in measuring its latency factor.

**Q.7: **All data sources are available and can be used to ensure full reproducibility. There is a review that the data on the figure illustration (Figure 2 and Figure 3) is too large so that it needs revision.

**A.7: **The figure illustration has used guidelines. 

**Q.8: **The conclusions drawn are supported by the results of data processing. Data processing requirements are adjusted to written objectives in an operational and measurable manner. 

**A.8: **I have added the LISREL analysis result in score to the "Care" indicator (EA 1a, 0.46<1.96), Critical (EA 3b, 0.64<1.96), and Local wisdom (EA 6b, 0.03<1.96) are not fulfilled the significance criteria.

**Q.9: **References are used both as a guide for the path of ideas (state of the arts) and discussion. Reference is up to date and applicable to the problem.

**A.9: **Thank you
